# Clinical Trials of Cancer Immunogene Therapies in Companion Animals: An Update (2017–2024)

**DOI:** 10.3390/vetsci12040329

**Published:** 2025-04-03

**Authors:** Gerardo C. Glikin, Liliana M. E. Finocchiaro

**Affiliations:** Unidad de Transferencia Genética, Área Investigación, Instituto de Oncología “Ángel H. Roffo”, Universidad de Buenos Aires, Av. San Martín 5481, Buenos Aires 1417, Argentina

**Keywords:** cancer, gene therapy, immunotherapy, companion animals, comparative oncology, clinical trials

## Abstract

This review summarizes the findings of veterinary clinical trials on immunogene therapy published between 2017 and 2024. Various tumor types, including melanoma (canine and feline), mastocytoma (canine), mammary adenocarcinoma (canine), osteosarcoma (canine), and sarcoid (equine), were treated using diverse strategies. Non-viral vectors were predominantly used to deliver genes encoding tumor-associated antigens, cytokines, or suicide enzymes. Among these non-viral methods, electrotransfer was the most commonly employed technique for introducing therapeutic genes into cells. Generally, these procedures resulted in minimal or no adverse side effects, and treated animals often showed significant improvements, such as enhanced quality of life, delayed or suppressed tumor recurrence or metastasis, and increased survival times. Some of these innovative approaches hold great potential as adjunct therapies to standard treatments. The promising outcomes from immunogene therapy studies in companion animals strongly support their application in veterinary oncology and provide valuable preclinical data (including safety assessments and proof-of-concept studies) for analogous human clinical trials.

## Simple Summary

Through the transfer of genetic information, immunogene therapy aims to increase tumor rejection in cancer patients by increasing the immunogenicity of certain tumor antigens as well as decreasing cancer tolerance mechanisms. Treating cancer-bearing veterinary patients can greatly accelerate translational research, providing benefits to both veterinary and human medicine. Recent findings have highlighted the safety and effectiveness of various immunogene therapy strategies. Expanding on our previous reviews, which explored advancements from 1996 to 2016, this updated review focuses specifically on veterinary cancer immunogene therapy, summarizing published studies in the field from 2017 to 2024.

## 1. Introduction

Nearly thirty years have passed since Quintin-Colonna and colleagues published their groundbreaking 1996 study [1] on ex vivo interleukin-2 immunogene therapy for feline fibrosarcoma and canine melanoma. In two previous reviews [2,3], we examined advancements in this field from its inception through to the end of 2016. The present review covers the developments published from January 2017 to December 2024. Since the beginning, most of the veterinary cancer gene therapy trials carried out have aimed to enhance the immune response against tumor cells.

The primary goal of immunotherapy is to enhance the body’s immune system to eliminate tumor cells and establish a long-lasting antitumor immune response, avoiding local invasion and the spread of metastatic disease. Among the different immunotherapeutic strategies, immunogene therapy strengthens the antitumor immune response by using genetic engineering techniques to introduce genes that usually encode tumor-associated antigens, cytokines, co-stimulatory molecules, and/or chemokines, into different kinds of patient’s cells in vivo or ex vivo (depending on the chosen approach) [4,5].

This review focuses on studies that have implemented strategies to actively stimulate a systemic antitumor immune response through the expression of transgenes, which, in the field of companion animal oncology, mostly encode either tumor antigens or cytokines.

## 2. Immunogene Therapy in Veterinary Oncology

What makes clinical trials of cancer immunogene therapy in companion animals significant? Despite progress in tumor treatment for both companion animals and human patients, there is still a critical need for novel therapeutic strategies to combat various malignancies, as current treatments often have limited effectiveness and cause significant side effects. Spontaneous tumors in companion animals serve as valuable translational models because pets exhibit relatively large body sizes, diverse genetic backgrounds, and intact immune systems. These models provide robust proof-of-concept evidence in a context comparable to human patient populations while simultaneously offering insights into toxicity and long-term efficacy. Currently, there is renewed interest in utilizing canine clinical trials to drive progress in cancer therapies [6,7,8].

Different gene therapy approaches were used in clinical studies against various types of tumors: from non-viral vectors such as “naked” plasmids, lipoplexes (cationic lipids/DNA complexes), non-replicative, and conditionally replicative viral vectors such as adenovirus, herpesvirus, poxvirus [2,3]. In some cases, physical methods such as jet injection, micro-seeding, and electrotransfer were also used to improve plasmid-mediated gene transfer.

As in our earlier reviews [2,3], we have focused exclusively on clinical data gathered from client-owned animals with naturally occurring tumors.

Table 1 displays twenty-six veterinary clinical trials reported from 2017 to 2024. They mainly involved non-viral gene transfer (19/26), sometimes enhanced by cationic lipids (2/26) and often by physical methods (15/26: 12 electrotransfer and 3 jet injection). In the remaining trials, viral vectors were used (7/26: 2 adenovirus, 2 vesicular stomatitis virus, 1 herpes simplex virus, 1 influenza virus, 1 poxvirus). Of the 26 veterinary clinical trials seen in Table 1, 23 correspond to canine patients, 2 to equines, and 1 to felines. Except for the feasibility/safety studies that covered different types of tumors (3 trials), those that focused on specific types include, in descending order of number of trials, canine melanoma (10), canine mastocytoma (4), canine mammary carcinoma (2), canine osteosarcoma (2), equine sarcoid (2), canine brain tumors (1), canine lymphoma (1), and feline melanoma (1). Regarding the types of trials, 9 were pilot studies, 5 were retrospective studies (of which 2 were controlled), and 12 were prospective studies (of which 2 were controlled and 4 compared with historical controls).

Early reports about feasibility and safety of autologous ex vivo immunogene therapies such as genetically modified dendritic cells [9] and chimeric antigen recombinant T cells (CAR-T) [10] or tumor infiltrating CAR-T cells (CAR-TILs) [11] were not included in this review.

## 3. Antigen Expression-Based Immunogene Therapy

### 3.1. Xenogeneic Tyrosinase

Spontaneous canine mucosal melanoma is a highly aggressive tumor of the oral cavity, digit/footpad, and muco-cutaneous junctions; it is chemo- and radio-resistant, does not respond well to treatment with conventional biological response modifiers, and shares similar metastatic phenotypes and site selectivity [2]. At the time of diagnosis, the disease is often metastatic and has an extremely poor prognosis because of rapid invasion of surrounding normal tissue and high likelihood of regional and distant metastasis early in the course of the disease. Its high metastatic rate and the inefficacy of current therapies warranted the investigation into novel therapies [12].

Preclinical and clinical studies have shown that xenogeneic DNA vaccination with tyrosinase gene family members can produce immune responses against melanoma, resulting in tumor rejection or protection and prolongation of survival. Conversely, syngeneic vaccination with orthologous DNA does not induce immune responses. Although tyrosinase may not seem to be a preferred target in amelanotic canine melanoma when determined by immunohistochemistry, other techniques showed significant tyrosinase overexpression in both melanotic and amelanotic melanomas [13].

The vaccination with plasmids carrying genes encoding tyrosinase family members demonstrated the ability to elicit both antibody and cytotoxic T cell responses leading to tumor rejection. This strategy offered a promising therapeutic approach and provided the basis for conducting the first trial of xenogeneic DNA vaccination in canine malignant melanoma using the human tyrosinase (hTyr) gene [14]. Following surgery, the vaccine was administered using a jet injection transdermal device (four biweekly doses followed by a booster every 6 months. Since this treatment was already approved by the US Department of Agriculture, the treatment is available, and additional studies were performed in diverse veterinary centers.

A retrospective study examined the outcomes of 69 canine oral melanoma patients with previous locoregional therapy treated with hTyr DNA vaccination [15]. A median survival time (MST) of 455 days, a figure comparable to earlier findings [16,17], was reported. Observing the responses in a group of 13 patients with macroscopic disease whose MST was 178 days, the authors proposed that the vaccine could serve as a palliative treatment for dogs with residual tumors or recurrence.

A new multi-institutional retrospective study offered new data about the clinical outcome of 131 dogs with oral melanoma (stages I to III) treated with hTyr DNA vaccination [18]. In this case, most dogs were also subjected to surgery and/or radiotherapy but not to chemotherapy. Again, a median survival time of 442 days was similar to earlier results [16,17], including the one described above [15]. As expected, a multivariable analysis indicated a negative correlation of clinical outcome with the stage of disease, while radiotherapy had a protective effect against tumor progression.

Even though a more recent retrospective small study focused on canine foot pad malignant melanoma included some patients (8/20) receiving hTyr DNA vaccination [19], there are very few data regarding its specific effects.

A small pilot study, comprising six oral melanoma canine patients, proposed DNA microseeding as an alternative delivery method for hTyr DNA vaccination [20]. This method was well tolerated with no significant toxicity detected and no signs of autoimmunity. Both local hTyr expression and humoral anti-human tyrosinase antibodies were detected. Further studies are necessary to assess the efficacy of this approach.

In general, the human tyrosinase DNA melanoma vaccine was well tolerated by canine patients [14,15,16,17,18,19,20,21]. Minimal adverse effects were described by owners, most being mild and self-limiting; the frequency of adverse reactions declined throughout the vaccination course. The lack of control groups and the diversity of concomitant treatments precluded the assessment of the clinical efficacy of the approach.

Similarly to dogs, malignant melanoma in cats is locally aggressive and highly metastatic, regardless of the primary site of origin [22,23]. The usual modalities, such as surgery and radiotherapy, often yield poor long-term results. Building on studies involving xenogeneic antigen vaccination for spontaneous canine malignant melanoma [21], research has reported the use of a transdermal, needle-free jet injection of a plasmid containing the hTyr gene. This plasmid’s expression as a melanoma xenoantigen was tested against feline melanoma [22].

In a retrospective study, the safety of the canine melanoma DNA vaccine following surgery in 24 feline patients was assessed [22]. The vaccine appeared to be well tolerated, with a low number of reported adverse events. Since most cats ultimately died from this disease, prospective controlled studies are essential to assess the immunogenicity and efficacy of this vaccine, originally developed for canine melanoma, when used to treat feline melanoma. The use of the hTyr DNA melanoma vaccine remains controversial, as studies have yielded mixed results regarding its effectiveness in providing benefits or extending lifespan. While the vaccine appears to be safe, there does not appear to be evidence that it improves outcomes when used [24].

### 3.2. Xenogeneic Chondroitin Sulfate Proteoglycan-4

Chondroitin sulfate proteoglycan-4 (CSPG4) serves as an early cell surface marker of tumor progression and is linked to enhanced migration, invasion, and proliferation of tumor cells [25]. In a previous trial involving a xenogeneic immunogene therapeutic approach, a human CSPG4 DNA-based vaccine had been evaluated for canine oral malignant melanoma [26]. This treatment was proposed as an adjuvant to surgery and transferred by periodic electro-vaccination with a plasmid carrying the hCSPG4 gene, allowing the assessment of its immunogenicity, safety, and therapeutic efficacy.

In a later prospective study with hCSPG4-encoded plasmid for II/III-staged CSPG4-positive oral canine malignant melanoma, 42 dogs were subjected to en bloc resection [27]. Twenty-nine patients were electro-vaccinated and the remaining nineteen dogs were assigned to the control group. The median survival time (MST) was 684 days for the vaccinated group and 200 days for the unvaccinated controls. Smaller dogs (<20 kg) displayed longer MST. Local relapse and lung metastatic rate diminished in vaccinated dogs (34.8 and 39%) with respect to unvaccinated controls (42 and 79%).

A retrospective study evaluated 82 dogs treated with adjuvant CSPG4-DNA electro-vaccination with oral malignant melanomas (stages I to IV) [28]. Both median survival times in dogs surgically treated with a curative intent (51 dogs) and those marginally excised only (31 dogs) (MST: 1333 vs. 470 days) as well as the corresponding disease-free intervals were significantly different (DFI: 324 vs. 184 days). On the other hand, the corresponding 63 non-vaccinated controls that received alternative adjuvant therapies displayed no significant differences in median survival time between dogs surgically treated with a curative intent (37 dogs) and those marginally excised only (26 dogs) (MST: 594 vs. 458 days), disease-free intervals were significantly different (DFI: 232 vs. 183 days). The authors concluded that a curative intent surgical approach, when feasible, is advisable in an attempt to prolong both the DFI and survival.

In a parallel retrospective study with 39 dogs treated with adjuvant CSPG4-DNA electro-vaccination with oral malignant melanomas, the authors [29] did not find significant relationship between leukocytes ratios and histological parameters, CSPG4 expression, excision margin status, age, tumor size, and clinical stage. Pretreatment neutrophil to lymphocyte (NLR) and lymphocyte to monocyte (LMR) ratios did not show a prognostic impact on the survival time of the entire population.

A high percentage of oral canine melanomas express the CSPG4 antigen [30]. Being a self-antigen, autologous CSPG4 is poorly immunogenic. To overcome this issue a chimeric human–canine CSPG4 gene (HuDo-CSPG4) was designed and inserted in a plasmid for DNA electro-vaccination. In a prospective study, 80 canine patients were included with surgically resected, CSPG4-positive, stage II–IV oral melanoma [31]. While 52 dogs received adjuvant HuDo-CSPG4 electro-vaccination, the remaining 28 were unvaccinated controls that only received conventional therapies. Dogs that received adjuvant vaccination demonstrated a significantly longer overall survival compared to the control group treated solely with conventional therapies. The median survival time (MST) was 653 days for the vaccinated group and 310 days for the control group. The implementation of this novel anti-CSPG4 mimicry strategy utilizing the hybrid HuDo-CSPG4 DNA vaccine proved to be safe and immunogenic, showing potential clinical benefits in extending survival.

Osteosarcoma represents approximately 85–90% of primary bone cancers in dogs. It is a common, highly metastatic cancer that predominantly affects large to giant breeds, primarily targeting the appendicular skeleton [32]. When adjuvant chemotherapy fails to control its disseminated form, there are no alternative treatment options available. Despite the surgical removal of the primary tumor before clinical detection of metastases, nearly all affected dogs eventually develop metastases in the lungs, bones, or other sites.

In a pilot veterinary study, 25 dogs with limb amputated, CSPG4-positive, stage I–III appendicular osteosarcoma were involved [33]. Patients also received standard chemotherapy to prevent the systemic spread of the disease. While 12 dogs received adjuvant HuDo-CSPG4 electro-vaccination, the remaining 13 were unvaccinated controls that only received conventional therapies. Dogs that received adjuvant vaccination demonstrated a significantly longer overall survival compared to the control group treated solely with conventional therapies. The median survival time (MST) was 484 days for the vaccinated group and 202 days for the control group. In addition, the disease-free interval (DFI) was 242 days for the vaccinated group and 160 days for the control group. In summary, the HuDo-CSPG4 vaccine has been shown to be safe and effective in inducing anti-CSPG4 immunity in dogs affected by osteosarcoma, leading to prolonged survival compared to control groups.

### 3.3. Other Antigens

Lymphoma is the most common hematopoietic cancer in dogs, accounting for approximately 15–20% of new canine cancer diagnoses [34]. The majority of these cases are malignant B-cell lymphomas, with diffuse large B-cell lymphoma (DLBCL) being the most prevalent subtype, often diagnosed at advanced stages (III–V) and typically exhibiting an aggressive clinical progression that necessitates prompt treatment. While multi-agent chemotherapy can achieve complete remission in some cases, the mortality rate for this neoplasm remains high.

A vaccine against dog telomerase reverse transcriptase (dTERT) was also proposed as a valid target for immunotherapy of diffuse large B-cell lymphoma [35]. The protocol, that includes a previous CHOP (cyclophosphamide, vincristine, doxorubicin, and prednisone) chemotherapy treatment, comprises an initial injection of an adenoviral vector containing the dTERT gene followed by periodic electro-vaccination boosts of a chimeric plasmid containing the dTERT fused at N-term with tissue plasminogen activator (TPA) leader sequence and at the C-term with B subunit of E. coli heat-labile enterotoxin (LTB). Seventeen canine patients with diffuse large B-cell lymphoma (DLBCL) were treated, and antibodies against the immunizing antigen were detected. The median survival time (MST) of 452 days observed in these patients was significantly longer than the 205 days recorded for control dogs treated only with COP chemotherapy (cyclophosphamide, vincristine, and prednisone) [36].

Horses and other equid species are commonly affected by skin tumors known as sarcoids, which are caused by bovine papillomavirus type 1 and/or 2 (BPV1, BPV2). Although sarcoids do not metastasize, they pose a significant health challenge due to their resistance to BPV1/2-mediated treatments and their tendency to recur in a more severe and widespread form after accidental or medical trauma [37].

Recombinant influenza (A and B) virus carrying the sequences of BPV1oncoproteins E6 and E7 were proposed as an immunogene therapy [38]. Intratumoral injections were applied to 29 equine patients bearing sarcoids. The treatment was safe and effective: 10 patients achieved complete responses and 10 partial responses at the end of the trial. Among the 29 individuals treated, 9 with severe conditions and a history of unsuccessful therapeutic attempts either failed to respond (6 cases) or exhibited only temporary improvements (3 cases). The fact that, in some cases, untreated lesions also displayed antitumor effects indicated a systemic antitumor response.

In unspayed female dogs, mammary tumors are the most frequently observed type, comprising roughly 50% of all tumors, with about half of these tumors being malignant [39]. The p62 protein (SQSTM1) is crucial for selective macroautophagy and functions as a central hub for various signal transduction pathways. Notably, while p62 is dispensable for normal tissue function, it is essential for tumor development and survival [40]. A study tested the effects of an intramuscular p62 plasmid DNA vaccine on mammary tumors in six dogs [41]. Locally advanced tumors either reduced in size (5/6) or stabilized (1/6) without any observed toxic side effects. Moreover, p62 treatment enhanced the presence of intratumoral T cells. All patients treated with p62 remain tumor- and metastasis-free and continued with a good quality of life four years after undergoing mastectomy. This pilot study suggests that p62 DNA treatment has the potential to reprogram the tumor stroma and may serve as an adjuvant in cancer therapies, exerting its effects directly or indirectly through immune responses.

CD40 ligand (CD40L, also known as CD154) is a powerful activator of antigen-presenting cells, such as dendritic cells (DCs). Additionally, it promotes the M1 phenotype and diminishes tumor infiltration by myeloid-derived suppressor cells. As a result, adding CD40L to the tumor microenvironment can provoke tumor inflammation and stimulate antitumor immune responses. AdCD40L is a replication-deficient adenovirus that encodes the human CD40L gene [42].

In a study of local AdCD40L treatment, 32 cases of mucosal canine melanoma (stages I to IV) were intratumorally injected [43]. In 20 cases, this was combined with cytoreductive surgery while the remaining 12 cases only received immunotherapy. Tumor tissue was infiltrated with T and B lymphocytes after treatment, suggesting immune stimulation. The best overall responses based on results of immunotherapy were 7 complete, 5 partial responses, 5 stable, and 2 progressive diseases. This treatment offered a good safety profile and a median survival of 285 days to dogs with melanoma.

## 4. Cytokine Expression-Based Immunogene Therapy

Only four types of cytokine genes were used in veterinary clinical situations from 2017 to 2024: interleukin-12, interleukin-2, interferon-β, and granulocyte–macrophage colony-stimulating factor. Usually, these genes were employed combined with other standard therapies such as chemotherapy, radiotherapy, molecular targets [2,3]. In some cases, a cytokine gene was combined with another cytokine gene, a suicide gene, or eventually inserted in a conditionally replicative oncolytic virus.

### 4.1. Interleukin-12 (IL-12)

IL-12 is viewed as an auspicious cytokine for boosting antitumor immune responses; nevertheless, recombinant IL-12 has exhibited substantial toxicity and limited effectiveness in early clinical trials. Recently, many approaches for delivering IL-12 to tumor tissues have been developed, such as modifying IL-12 structure, employing viral vectors, non-viral vectors, and cellular vectors [44]. As one of the most potent proinflammatory cytokines, it was initially investigated for this purpose. However, initial murine and human studies in which IL-12 was administered systemically resulted in dangerous immunotoxicity associated with off-target immune activation [45].

Gene electrotransfer (GET) was the method of choice for delivering plasmids carrying the IL-12 gene into cells in vivo. In a pilot study, nine dogs with various spontaneous cancers underwent three consecutive intratumoral hIL-12 GET sessions to evaluate their clinical, immunological, and anti-angiogenic effects [46]. Both serum and tumor tissue showed temporary increases in canine interferon-γ (IFN-γ) and hIL-12 levels. Although intratumoral IL-12 GET demonstrated some anti-angiogenic and immunostimulatory activities along with transient objective responses, it did not yield clinically significant results. Sustained tumor regression could not be achieved during the trial. The therapy was confirmed to be safe, with no severe side effects observed.

The effects of combining three successive intratumoral hIL-12 gene electrotransfer treatments with metronomic cyclophosphamide were evaluated in six dogs with various spontaneous tumors [47]. This combination therapy resulted in tumor erythema and swelling, a transitory rise in IL-12 levels, and sustained increases in IFN-γ and thrombospondin-1, accompanied by an uninterrupted reduction in vascular endothelial growth factor (VEGF). The treatment improved body weight and quality of life while slowing tumor progress in the majority of patients (4 out of 6).

Mast cell tumors (MCTs) are among the most common cutaneous neoplasms in dogs, representing up to 21% of all canine skin tumors. While most cases can be effectively treated with local therapy, some exhibit biologically aggressive behavior, leading to local recurrence or metastasis [48].

Peritumoral hIL-12 gene electrotransfer combined with electro-chemotherapy (using cisplatin and/or bleomycin) was evaluated in 18 canine patients with MCTs [49]. This combination therapy achieved significant local tumor control within one month without causing side effects. The complete response rate rose to 72% over the observation period. Serum hIL-12 and/or IFN-γ levels were detectable in 78% of patients, demonstrating systemic immune activation. This study highlighted the high antitumor efficacy of this combined treatment in preventing recurrences and distant metastases while establishing its safety and feasibility.

As a follow-up to this study, histopathological analysis was performed on samples from 11 patients. The analysis confirmed that the combined electro-chemotherapy and IL-12 gene electrotransfer effectively triggered a cellular immune response against neoplastic cells. This was characterized by the recruitment of T-lymphocytes and macrophages, along with fibrotic proliferation and a reduction in microvessels [50].

In an additional trial, eight dogs (seven bearing mast cell tumors and one neurofibrosarcoma), were subjected to the combined i.v. bleomycin or i.t. cisplatin electro-chemotherapy and cIL-12 gene electro-transfer [51]. Dynamic contrast-enhanced ultrasound (DCE-US) serial examinations were conducted: before therapy, shortly after therapy, and long-term post-therapy. Tumors that achieved complete responses exhibited significantly lower capillary perfusion and perfusion heterogeneity compared to those that did not achieve complete responses. DCE-US proved to be a safe procedure, with no adverse effects observed following repeated administration. Moreover, it can provide a straightforward and repeatable method for assessing tumor perfusion during and after treatment. This diagnostic method could serve as a predictor of tumor responses, aiding in the evaluation of therapeutic options.

A prospective study recruited 77 dogs with spontaneous mast cell tumors (MCTs) and assigned them to three groups: one treated with a combination of ECT + GET p.t. (29 dogs), the second with the combination of ECT + GET i.t. (30 dogs), and the third with ECT alone (18 dogs) [52]. The results showed that local tumor control was significantly better in the ECT + GET i.t. group than in the ECT + GET p.t. or ECT groups. In addition, disease-free interval (DFI) and progression-free survival (PFS) were significantly longer in the ECT + GET i.t. group than in the other two groups. The data on local tumor response, DFI, and PFS were consistent with an increased percentage of circulating antitumor immune cells in the ECT + GET i.t. group, which also indicated the induction of a systemic immune response. No unwanted severe or long-lasting side effects were observed.

In a later trial involving 48 canine patients with 86 mast cell tumors (MCTs), blood samples were collected before ECT + GET i.t. treatment, and again at one- and six-months post-treatment, to identify trustworthy biomarkers predicting treatment response [53]. An increase in plasma nucleosome levels and serum lactate dehydrogenase (LDH) activity one month after treatment was related to a more robust local response, such as necrosis and swelling. These biomarkers displayed promise as early indicators of treatment success.

When assayed in canine oral malignant melanoma, a similar approach involved a combination of cytoreductive surgery, ECT with bleomycin, and p.t. cIL-12 GET [54]. While three dogs did not respond to treatment, six dogs displayed transient objective responses (4 CR, 2 PR) that later derived in PD; MST was 180 days. The observed reduction in circulating regulatory T cells (Treg) may be the result of a systemic antitumor response triggered by cIL-12 GET. This alternative combination therapy for advanced canine melanoma showed a good safety profile.

In general terms, candidate genes for electrotransfer, the delivery site, the dosage of plasmids encoding these genes, and the dose of chemotherapeutic agents require further investigation [55]. While peritumoral delivery of a plasmid encoding IL-12 via GET has shown promising results in treating various tumor types in dogs, the efficacy of intratumoral plasmid application remains to be determined.

Certain breeds of pet dogs naturally and sporadically develop high-grade gliomas. The most aggressive of these tumors resist the available limited treatment options, resulting in poor outcomes with median survival times of about 2 months in dogs [56]. A new approach proposed the combination of intracranial surgery, oncolytic virotherapy, and immunogene therapy [57]. Following surgery, a targeted conditionally replicating oncolytic virus carrying the hIL-12 gene was infused in the cavity. The trial included 21 dogs: 13 had high-grade gliomas, 5 had low-grade gliomas, and 3 were undetermined. The MST was 151 days and no significant adverse events were reported. The reported MST for symptomatic treatments was 65 days [58]. In a parallel study, it was demonstrated that this oncolytic herpes virus vector modulates the tumor-immune microenvironment in canine glioma patients [59]. All these early data support further trials for assessing the efficacy of the approach.

### 4.2. Interleukin-2 (IL-2)

IL-2 is a potent pleiotropic cytokine involved in exerting dual functions of driving effector T cell expansion and differentiation as well as mediating peripheral tolerance by inducing regulatory T cells. Due to its capacity to activate and expand cytotoxic effector cells, IL-2 immunotherapy has been examined in numerous clinical trials [60]. To overcome the systemic toxicity of this cytokine, several novel engineering strategies are being developed for IL-2 based cancer treatments, including approaches such as immunogene therapy.

The canarypox virus vector ALVAC bearing the feline IL-2 gene was tested for treating equine sarcoids [61]. In a prospective proof of concept study, 14 horses received periodical intratumor injections of the vector. The best responses were 7 CR, 5 PR. The median time to best response was 211 days. Adverse events were minimal. The results offer preliminary evidence that ALVAC-fIL2 is a safe and effective treatment for sarcoid tumors in horses.

Even though IL-2 gene was initially proposed as a single immunogene treatment, it was usually combined with other cytokine genes or other modalities like chemotherapy, ECT, or suicide gene therapy.

In a retrospective controlled trial, 30 dogs with inoperable stage III--IV oral canine melanoma were treated with bleomycin ECT (the drug i.v injected) [62]. One-third of these dogs also received GET with two different cytokines: proximal intra-tumor cIL-2 and distal intramuscular cIL-12. Both groups showed similar local response rates and overall survival times. However, progression-free survival (PFS) resulted significantly better in the ECT + GET group (180 d) than in the ECT group (120 d). Both groups demonstrated comparable local response rates and overall survival times. However, the ECT + GET group showed significantly improved progression-free survival. With AEs between low and mild, a larger trial could help to assess the efficacy of the immune systemic effects mediated by the expressed cytokines for metastatic disease control.

### 4.3. Interferon-β (IFN-β)

Type I interferons (IFNs) have been demonstrated to inhibit tumor growth directly and indirectly by acting upon tumors and immune cells, respectively. Furthermore, accumulating evidence indicates that endo- and exogenously enhancing type I interferons have a synergistic effect on antitumor immunity [63]. Interferon-β (IFN-β) exhibits anti-proliferative, anti-angiogenic, and immunomodulatory properties. It was one of the earliest cytokines to receive approval for clinical application. Despite its clinical efficacy, the continuation of IFN-β therapy is frequently hindered by its intrinsic systemic toxicity.

In a pilot assay, a recombinant vesicular stomatitis virus (VSV) carrying the canine or human IFN-β and the sodium–iodide symporter (NIS): VSV-cIFNβ-NIS and VSV-hIFN-NIS [64]. Ten dogs with different tumors were intravenously injected with the vector (5 and 5). The feasible safety of repeated intravenous VSV-IFNβ-NIS administration was demonstrated and some evidence of potential efficacy was supported by the 2 PR and 3 SD outcomes. In the context of this assay and the next one, the NIS gene was not considered for therapeutic purposes.

As a continuation of this assay, a new prospective trial with the recombinant vesicular stomatitis virus VSV-cIFNβ-NIS for canine appendicular osteosarcoma was performed [65]. In a neoadjuvancy setting, the recombinant oncolytic virus was intravenously injected 10 days before amputation. All dogs received standard carboplatin chemotherapy 14 days after amputation. Twenty-two dogs received systemic neoadjuvant intravenous VSV-cIFNβ-NIS and six received placebo. MST of treated patients was not significantly different from that of historical controls: most of them died due to disease progression. However, there was a group of seven long-term survivors (survival >479 days) that died of unrelated causes or were still alive at the end of this study. With a good safety profile, this study proved that pre-treatment immune infiltration is associated with longer overall survival.

### 4.4. Granulocyte–Macrophage Colony-Stimulating Factor (GM–CSF)

GM–CSF is a cytokine that drives the generation of myeloid cell subsets including neutrophils, monocytes, macrophages, and dendritic cells in response to stress, infections, and cancers [66]. By modulating the functions of innate immune cells that serve as a bridge to activate adaptive immune responses, GM–CSF globally impacts host immune surveillance under pathologic conditions. While little GM–CSF induces the appropriate production of innate immune cells and subsequent activation of adaptive anti-cancer immune responses, too much GM–CSF can exhaust immune cells and promote cancer growth.

Thirty-six canine mammary carcinoma patients underwent injections of lipoplexes carrying canine interferon-β (cIFN-β) and HSV-thymidine kinase (HSV-tk) genes ombined with ganciclovir (GCV) into the tumor bed directly following surgery [67]. Subsequently, patients received periodic subcutaneous injections of lipoplexes containing human granulocyte–macrophage colony-stimulating factor and interleukin-2 genes, mixed with allogeneic mammary carcinoma extracts. This combined therapeutic approach was safe and well-tolerated. Notably, among the 26 patients who underwent complete surgery, only 2 experienced local relapse, and none of the 29 stage II and III patients developed distant metastases, suggesting effective local and systemic antitumor activity. The most promising outcome was the extended survival times: 22 patients survived over 1 year, with 13 exceeding 2 years, and 4 surpassing 3 years, all while maintaining a good quality of life. In five additional patients with local disease, the inclusion of an extra HSV-tk/GCV plus cIFN-β gene treatment demonstrated local antitumor activity, resulting in four objective responses (one complete and three partial) and one case of stable disease. These results highlight the potential of this therapeutic strategy and support further studies with a control arm to validate its efficacy.

In a prospective controlled trial 364 canine malignant melanoma patients received after complete or partial surgery: (i) a combined treatment that involves intra/peri-tumoral injection of lipoplexes containing the HSV-tk/r suicide gene, ganciclovir, canine interferon-β gene and bleomycin intra and/or (ii) a subcutaneous vaccine composed by canine melanoma cell extract and lipoplexes containing hIL-2 and hGM-CSF genes [68]. The control group was 173 dogs treated only with surgery. The adjuvant treatment (AT) group was divided into three groups: (i) complete surgery plus vaccine (CS-V), (ii) complete surgery plus combined treatment (CS-CT), and (iii) partial surgery plus combined treatment (PS-CT). Compared to the surgery-only control group (So), both CS-CT and CS-V significantly improved outcomes. The proportion of patients free from local disease increased from 20% to 89% (CS-CT) and 74% (CS-V), while those without distant metastases (M0) increased from 45% to 87% (CS-CT) and 84% (CS-V). Although less effective than CS groups, the PS-CT arm still showed significantly better control of metastatic disease (M0: 80%) compared to So (M0: 44%). Adjuvant therapy also led to substantial increases in overall survival, with a 9.3-fold (CS-CT), 6.5-fold (CS-V), and 5.4-fold (PS-CT) improvement over their respective So controls. In general, AT transformed melanoma from a lethal disease into a chronic condition, with 70% of CS-CT, 51% of CS-V, and 14% of PS-CT patients succumbing to non-melanoma-related causes. These treatments successfully delayed or prevented post-surgical recurrence and metastasis, improved disease-free and overall survival rates, and preserved quality of life.

A significant increase in overall survival was observed in the PS-CT and CS-CT patients (415 and 880 days, respectively) compared to the survival rates in a similar trial without BLM (323 and 704 days, respectively) [69], supporting the use of concomitant local chemotherapy to achieve a better outcome.

In a variant of the treatment combining surgery with local suicide gene system plus IFN-β gene plus bleomycin, and subcutaneous vaccines stimulated by cytokine genes [68], the suicide gene system was replaced by locally applied 5-fluorouracil chemotherapy. Preliminary encouraging data from our team indicate safety and disease control in the treatment of various solid tumors as indicated by the following median survival time values: canine melanoma (844 d, *n* = 186), canine fibrosarcoma (500 d, *n* = 20), canine osteosarcoma (238 d, *n* = 11), feline melanoma (491 d, *n* = 10), feline squamous cell carcinoma (474 d, *n* = 47) feline fibrosarcoma (545 d, *n* = 43), and equine melanoma (all alive, mean time under treatment > 1024 d, *n* = 14).

Beyond the encouraging results of the pilot studies and the studies without proper control arms, four prospective controlled trials [27,31,52,68] and one retrospective [28] demonstrated a statistically significant improvement in the antitumor effects (including in some cases extended survival) while maintaining a good quality of life. These findings provide strong evidence for further advancements in immunogene therapy.

Regarding the versatility of the methodology, non-viral systems involving IL-12 GET, CSPG4 GET, and the suicide gene system plus IFN-β (combined with cytokine-enhanced vaccines), have shown encouraging results in different types of canine tumors: mastocytoma and melanoma for the IL-12 gene [52,54], melanoma and osteosarcoma for the CSPG4 gene [31,33], and melanoma and mammary carcinoma for the suicide gene system [67,68].

## 5. Conclusions

Some studies were focused on validating gene transfer and expression methods by treating few patients with different kinds of tumors in the same study where the heterogeneity and the absence of control groups precluded conclusions about the efficacy of the treatment. Nevertheless, these studies were valuable as feasibility, proof of concept, and safety assessment.

On the other hand, larger studies involving higher numbers of patients with the same kind of tumor, often with suitable control groups, were able to generate statistically significant data to validate the efficacy of the proposed treatments.

For biosafety reasons (concerning both animals and humans), as well as the higher cost of viral vector production, most veterinary cancer gene therapy protocols have relied on non-viral vectors, with electrotransfer being the predominant delivery method from 2017 to 2024.

To date, more than 2054 clinical protocols for gene therapy for cancer have been registered [70], resulting in 20 treatments approved worldwide for marketing, according to the American Society of Gene and Cell Therapy (ASGCT). In veterinary oncology, the situation is much more modest, with around a hundred publications on the subject and only two treatments approved for commercialization: a plasmid vector with the human tyrosinase gene as a xenoantigen for canine melanoma (Oncept™ Canine Melanoma Vaccine, Duluth, GA, USA) and a canarypox virus vector with the feline interleukin-2 gene for feline fibrosarcoma (Oncept IL-2™).

Almost three decades have passed since the first report with clinical results of the application of genetic immunotherapy in companion animals [1]. Despite proposals for the implementation of veterinary clinical protocols for cancer therapies with a translational approach [6,7,8,71], the treatments approved so far for humans were not previously tested in companion animals.

On the other hand, some complex treatment approaches for autologous that involve genetic modifications of ex vivo cells, first went through clinical studies in humans than in companion animals. Although they may have high translational value, these technologies are currently very expensive to be applied in veterinary medicine.

Oncolytic virotherapy is emerging as a new tool against cancer in companion animals [72,73]. The update of those approaches that only used suicide genes and/or oncolytic viruses in companion animals would deserve a separate review.

In veterinary medicine, non-viral vectors probably offer a better opportunity to treat animal tumors in a more affordable way for owners. Greater investment in time and effort will be necessary to validate the results in prospective controlled studies and ultimately offer immunogene therapy to more patients who currently lack effective treatment options.

**Table 1:** Cancer immunogene therapy veterinary clinical trials 2017–2024.

#	Genes	Tumors	Vectors	Modes	Main Results	1st Authors/Year/Main Country
1	htyr	MEL	Plasmid	t.d. jet-injection + SX ± RX ± CHT	Retrospective study stages I–III OMM (*n* = 56): MST = 455 d; DFI = 222 d.	Verganti et al., 2017, UK [15]
2	htyr	MEL (feline)	Plasmid	t.d. jet-injection + SX ± RX ± CHT	Retrospective study (*n* = 24). Manageable post-vaccination AEs: 11%. Safety verified. 42% died of unrelated causes or still alive.	Sarbu et al., 2017, USA [22]
3	htyr	MEL	Plasmid	i.d. micro-seeding njection + SX	Pilot study stages I–IV (*n* = 6): 3 NED (2 oral, 1 dermal MEL; survival > 1 year), 3 PD (2 oral, 1 dermal MEL). No significant AEs.	Zuleger et al., 2017, USA [20]
4	hIL-12	MCT	Plasmid	p.t. GET + i.t. cisplatin or i.t. bleomycin ECT	Prospective study. Stages I–III (*n* = 18): 13 CR, 2 PR, 1 SD, 2 PD. No major AEs.	Cemazar et al., 2017, Slovenia [49]
5	hIL-12	ADC, FSA, MEL, OSA, SCH	Plasmid	i.t. GET + metronomic p.o. cyclophosphamide CHT	Pilot study (*n* = 6): Combined therapy slowed down tumor progression and improved QoL. No significant unwanted side effects.	Cicchelero et al., 2017, Belgium [47]
6	hIL12	FSA, MAC, MCT, OSA, SCC, SCH	Plasmid	i,t, injection	Pilot study (*n* = 9): No significant clinical benefits. Safety studies and demonstration of immunogenic and anti-angiogenic effects.	Cicchelero et al., 2017, Belgium [46]
7	hCSPG4	MEL	Plasmid	SX and i.m. GET	Prospective controlled study. Surgically resected Stage II/III, LN (-) OMM, VAX (*n* = 23)/CTR (*n* = 19): MST: VAX 684 d/CTR 220 d DFI: VAX 477 d/CTR 180 d Safety and immunogenicity confirmed.	Piras et al., 2017, Italy [27]
8	cIFNβ + NIS or hIFNβ + NIS	ADC, MMA, LYP, MEL, OSA	Recombinant oncolytic vesicular stomatitis virus: VSV-IFNβ-NIS	i.v. injection	Pilot study. (*n* = 10): 2 PR, 5 SD, 3PD. Low or mild/transient AEs. Safety and preliminary evidence of efficacy.	Naik et al., 2018, USA [64]
9	HSV-tk + cIFN-β hIL-2 + hGM-CSF	MAC	Plasmid lipoplexes	SX and i.t. (SG+ GCV+IFN-β) and s.c. [(hIL-2+ hGM-CSF)+(TV)]	Prospective study Stages II to IV: Combined treatment (*n* = 36). Complete surgery arm (*n* = 26): Local disease-free patients: 92%. Metastasis-free patients: 89%. MST = 876 d; metastasis-free MST > 1498 d. Partial surgery arm (*n* = 10). MST = 241 d.	Finocchiaro et al., 2018, Argentina [67]
10	cTERT	LYP	Recombinant adenovirus and plasmid	i.m virus. injection and i.m. plasmid GET+CHOP-CHT	Prospective study (*n* = 17): No significant AEs. Improved MST = 452 d as compared with previous historical (*n* = 21) COP-CHT controls MST = 205 d.	Impellizieri et al., 2018, Italy [35]
11	cIL-12	MEL	Plasmid	PSX/p.t. GET + i.v. bleomycin ECT	Prospective study. Stages I--III OMM (*n* = 9): 4 CR, 2 PR, 3 PD. MST = 180 d. No major AEs.	Milevoj et al., 2019, Slovenia [54]
12	hIL-2 + hGM-CSF cIFN-β + HSV-tk	MEL	Plasmid lipoplexes	SX and i.t. injection (SG+ GCV+IFN-β+ bleomycin) and s.c. [(hIL-2+hGM-CSF)+(TV)]	Prospective controlled study. Stages I–IV: Combined treatment (*n* = 210)/Surgery controls (*n* = 173). Complete surgery arms: Local disease-free patients: 89%/20%. Metastasis-free patients: 89%/45%. MST > 1896 d/99 d. Metastasis-free MST > 1896 d/120 d. No significant AEs.	Finocchiaro et al., 2019, Argentina [68]
13	hp62	MAC	Plasmid	i.m. injection followed by mastectomy	Pilot study. (*n* = 6: 3 solid and 3 tubo-papillary mammary carcinomas): 5 PR, 1 SD. Good QoL > 4 years after surgery.	Venanzi et al., 2019, Italy [41]
14	htyr	MEL	Plasmid	t.d. jet-injection + SX ± RX	Retrospective study, stages I–III (*n* = 131): MEL-MST = 510 d/All causes MST 442 d.	Turek et al., 2020, USA [18]
15	hIL-12	ODG, ACT, GBM	Recombinant oncolytic HSV-1 virus: M032	i.c inoculation.	Prospective study (*n* = 21): MST = 151 d. No significant AEs and dose-limiting toxicities.	Omar et al., 2021, USA [57]
16	hCSPG4	MEL	Plasmid	en bloc SX + i.m. GET	Retrospective controlled study Stages I–IV: En bloc SX (*n* = 51): MST = 1333 d; DFI = 324 Marginal SX (*n* = 31): MST = 470 d; DFI = 184.	Giacobino et al., 2021, Italy [28]
17	cIL-12	MCT	Plasmid	i.t. GET + i.t. cisplatin or i.v. bleomycin ECT	Pilot study (*n* = 8) 12 tumors (11 MCT, 1 NFS): 7 CR,2 PR, 3 PD. Differences in DCE-US parameters between tumors with CR and non-CR tumors were found.	Brloznik et al., 2021, Slovenia [51]
18	CD40L	MEL	Recombinant adenovirus: AdCD40L	i.t. injection ± SX	Pilot study (*n* = 32): MTS = 285 d MTS (+SX) = 448 d MTS (-SX) = 80 d, 7 CR, 5 PR, 5 SD, 2 PD, 13 MR. Transient mild AEs. Low toxicity.	Saellstrom et al., 2021, Sweden [43]
19	BPV E6/E7	SAR (equine)	Recombinant influenza virus: iNSA/E6E7^equ^ and iNSB/E6E7^equ^	i.t. injection	Prospective study (*n* = 29): 10 CR, 10 PR, 9 PD. Efficacy and safety were proven.	Jindra et al., 2021, Austria [37]
20	fIL-2	SAR (equine)	Recombinant canary pox virus. ALVAC-fIL2	i.t. injection	Pilot study (*n* = 14): 7 CR (50%), 5 PR (35%), 1 SD (7%), and 1 PD (7%) median time to best response = 211 d. Minimal AEs.	Saba et al., 2022, USA [61]
21	HuDo-CSPG4	MEL	Plasmid	en bloc SX + i.m. GET	Prospective controlled study. Stages II--IV: VAX Treated (*n* = 52): MST = 653 d Control (*n* = 28) MST = 310 d. Safe and effective.	Riccardo et al., 2022 Italy [31]
22	cIL-2 + cIL-12	MEL	Plasmid	i.t cIL-2 GET i.m.cIL-12 GET + bleomycin ECT	Retrospective controlled study. Stages III–IV GET + ECT (*n* = 10): MST = 165 d/PFS = 165 d. ECT (*n* = 20): MST = 180 d PFS = 120 d.	Tellado et al., 2023, Argentina [62]
23	cIL-12	MCT	Plasmid	i.t or p.t. GET+ i.t. cisplatin or i.v. bleomycin-ECT	Prospective controlled study: ECT (*n* = 18): CR = 69%; ECT + GETp.t. (*n* = 29): CR = 88%; ECT + GETi.t. (*n* = 30): CR = 94%. DFI and PFS: ECT + GETi.t. > ECT + GETp.t. ≈ ECT.	Lampreht et al., 2023, Slovenia [52]
24	HuDo-CSPG4	OSA	Plasmid	i.m.HuDo-CSPG4 + limb amputation + i.v. carboplatin-CHT	Pilot study: VAX + CHT treated (*n* = 12): MST = 484 d DFI = 242 d; historical CHT controls (*n* = 13): MST = 202 d; DFI = 160 d.	Tarone et al., 2023, Italy [33]
25	cIFNβ + NIS	OSA	Recombinant oncolytic vesicular stomatitis virus: VSV-cIFNβ-NIS	i.v. rec virus + i.v. carboplatin CHT	Prospective study (*n* = 28): 35% survival > 479 d. Safety documented.	Makielski et al., 2023, USA [65]
26	cIL-12	MCT	Plasmid	i.t GET + i.t./i.v. bleomycin ± i.t. cisplatin ECT	Prospective study (*n* = 48): after 1 month: CR = 43%; PR = 41%; PD = 0. After 6 months: CR = 77%; PR = 12%; PD = 6%. Two possible efficacy markers were defined.	Vilfan et al., 2024, Slovenia [53]

Clinical trials appear ordered according to their publication date. Unless otherwise indicated all tumor types are canine. All gene transfers were performed in vivo. TUMORS: ACT, astrocytoma; ADC, adenocarcinoma; FSA, fibrosarcoma; GBM, glioblastoma; LYP, lymphoma, MAC, mammary adenocarcinoma; MCT, mast cell tumor; MEL: melanoma; MMA, multiple mieloma; NFS, neurofibrosarcoma; ODG, oligodendroglioma; OMM, oral malignant melanoma; OSA, osteosarcoma; SAR, sarcoid; SCC, squamous cell carcinoma; SCH, schwannoma//GENES: BPV E6/E7, bovine papilloma virus E6/E7; CD40L, CD40 ligand; CSPG4, chondroitin sulfate proteoglycan-4; GM-CSF, granulocyte macrophage colony-stimulating factor; HSV-tk, herpes simplex thymidine kinase; IFN-β, interferon-β; IL-12, interleukin-12; IL-2, interleukin-2; NIS, sodium-iodide symporter; p62, protein 62; TERT, telomerase reverse transcriptase; tyr, tyrosinase//GENE PREFIXES: c, canine; f, feline; h, human; HuDo, human-dog chimeric//RESPONSES: AEs, adverse events; CR, complete response; CTR, control; DFI: disease-free interval, MR, mixed responses; MST, median survival time; NED, no evidence of disease; PD, progressive disease; PR, partial response; QoL, quality of life; SD, stable disease//TREATMENTS: CHT, chemotherapy; COP, cyclophosphamide, vincristine, and prednisone chemotherapy; CHOP, cyclophosphamide, vincristine doxorubicin and prednisone chemotherapy; ECT, electro-chemotherapy; GCV, ganciclovir; GET, gene electrotransfer; RX, radiotherapy; SG, suicide gene; SX, surgical excision; TV, tumor vaccine; VAX, vaccinated//ROUTES: i.c., intracranial; i.d, intradermal; i.m., intramuscular; i.t., intratumoral; p.o., oral; p.t., peritumoral; s.c., subcutaneous; t.d, transdermal//DCE-US, dynamic contrast-enhanced ultrasound//d, days.

## Data Availability

Not applicable.
